# Powers, engagements and resultant influences over the design and implementation of medicine pricing policies in Ghana

**DOI:** 10.1136/bmjgh-2021-008225

**Published:** 2022-05-18

**Authors:** Augustina Koduah, Leonard Baatiema, Irene A Kretchy, Irene Akua Agyepong, Anthony Danso-Appiah, Anna Cronin de Chavez, Timothy Ensor, Tolib Mirzoev

**Affiliations:** 1 Pharmacy Practice and Clinical Pharmacy, University of Ghana School of Pharmacy, Legon, Accra, Ghana; 2 Health Policy, Planning & Management, University of Ghana School of Public Health, Legon, Accra, Ghana; 3 Dodowa Health Research Centre, Ghana Health Service, Accra, Greater Accra, Ghana; 4 Faculty of Public Health, Ghana College of Physicians and Surgeons, Accra, Greater Accra, Ghana; 5 Epidemiology and Disease Control, University of Ghana School of Public Health, Legon, Accra, Ghana; 6 Global Health and Development, London School of Hygiene & Tropical Medicine, London, London, UK; 7 Nuffield Centre for International Health and Development, University of Leeds, Leeds, UK

**Keywords:** Health policy, Health systems

## Abstract

**Introduction:**

Universal availability and affordability of essential medicines are determined by effective design and implementation of relevant policies, typically involving multiple stakeholders. This paper examined stakeholder engagements, powers and resultant influences over design and implementation of four medicines pricing policies in Ghana: Health Commodity Supply Chain Master Plan, framework contracting for high demand medicines, Value Added Tax (VAT) exemptions for selected essential medicines, and ring-fencing medicines for local manufacturing.

**Methods:**

Data were collected using reviews of policy documentation (n=16), consultative meetings with key policy actors (n=5) and in-depth interviews (n=29) with purposefully identified national-level policymakers, public and private health professionals including members of the National Medicine Pricing Committee, pharmaceutical wholesalers and importers. Data were analysed using thematic framework.

**Results:**

A total of 46 stakeholders were identified, including representatives from the Ministry of Health, other government agencies, development partners, pharmaceutical industry and professional bodies. The Ministry of Health coordinated policy processes, utilising its bureaucratic mandate and exerted high influences over each policy. Most stakeholders were highly engaged in policy processes. Whereas some led or coproduced the policies in the design stage and participated in policy implementation, others were consulted for their inputs, views and opinions. Stakeholder powers reflected their expertise, bureaucratic mandates and through participation in national level consultation meetings, influences policy contents and implementation. A wider range of stakeholders were involved in the VAT exemption policies, reflecting their multisectoral nature. A minority of stakeholders, such as service providers were not engaged despite their interest in medicines pricing, and consequently did not influence policies.

**Conclusions:**

Stakeholder powers were central to their engagements in, and resultant influences over medicine pricing policy processes. Effective leadership is important for inclusive and participatory policymaking, and one should be cognisant of the nature of policy issues and approaches to policy design and implementation.

**WHAT IS ALREADY KNOWN ON THIS TOPIC**

- Design and implementation of any health policies involves multiple stakeholders with varied powers, roles, interests and agendas, though little is known about stakeholder roles and engagement in medicines pricing policies in low-income and middle-income countries.

**WHAT THIS STUDY ADDS**

- In Ghana, stakeholder engagements across four national medicine pricing policies were generally high-to-medium with strong leadership from the Ministry of Health but limited engagements from the private sector and local health workers.
- Stakeholder power was central to their engagements and resultant influences over policy design and implementation. All these were also affected by the nature of policy issue and approaches to policymaking.

**HOW THIS STUDY MIGHT AFFECT RESEARCH, PRACTICE AND/OR POLICY**

- Medicines pricing policies can be improved by: identifying key stakeholders and considering their experiences, perspectives and expectations; greater understanding of the nature of a policy issue and approaches to policy design and implementation and developing feasible and context-specific ways of enhancing stakeholder engagements within complex policy processes.

## Introduction

Improving availability and affordability of essential medicines is critical to achieving better health outcomes,^1 2^ and requires effective design and implementation of medicines pricing policies.^3–5^ Policymaking typically involves multiple stakeholders with varying roles, agendas and interests.^6 7^ Stakeholders are ‘…actors (individuals or organizations) who have interest in the issue under consideration, who are affected by the issue, or who—because of their position—have or could have an active or passive influence on the decision-making and implementation processes’.^8^ Stakeholder engagements in policymaking reflect their interests, and together with their exercise of power, determine their influences over policy decisions.^6 8–11^ Understanding these can reveal how policies are framed, whether and how policy implementation occurs and how all these can be improved. For example, understanding who led, who contributed and who were excluded from the policy design, can reveal how policy agenda for medicines pricing was determined and what evidence may have informed policy decisions; and the rationale for regulating (or not) medicines prices. This knowledge can inform effective interventions in regulating and reducing pricing of medicines, contributing to improved access to medicines.

Guidance is available on conducting stakeholder analyses^8 10^ and studies examined stakeholder roles in health insurance systems,^12^ health research priority-setting^13^ and provider payment systems.^14^ However, we found no publications from low-income and middle-income countries (LMICs) which examined stakeholder roles in design and implementation of medicines pricing policies. This paper contributes to bridging this knowledge gap by reporting our stakeholder analysis from four medicines pricing policies from Ghana. We answer the following question: *what were the roles, engagements and powers of different stakeholders, and how did these translate into stakeholder influences over the design and implementation of four medicine pricing policies in Ghana?* In doing so, we also enhance the understanding of processes of designing and implementing medicines pricing policies, although our primary focus remains on the stakeholder analysis. We anticipate this paper to be of interest and relevance to policymakers, practitioners and academics, who are interested and engaged in, understanding stakeholder roles and influences for improving medicines pricing policies and access to essential medicines, and ensuring effective design and implementation of health policy processes more generally.

### Four medicines pricing policies in Ghana

The Ghanaian health sector has a predominately, publicly administered and delivered services model, although with growing private sector participation in service provision. In addition to policy design and formulation, the Ministry of Health (MoH) has the mandate to coordinate activities within the health sector and authority over health policy processes. In doing so, the MoH works with its implementing agencies such as Ghana Health Service and National Health Insurance Authority (NHIA), as well as other government and non-government actors.^15^

Ghana’s pharmaceutical industry is the second largest in West Africa and total market value was estimated at $586 million in 2019.^16^ The pharmaceutical industry is the key component of the health sector, and its performance is driven by the health sector demand for medicines. The main pharmaceutical policy priorities are to ensure access to affordable, efficacious and safe medicines for everyone, strengthen local pharmaceutical industry and improve supply chain.^17^ Yet, medicine prices in Ghana are considered to be high, and several interventions were introduced to reduce price build-ups along the supply chain and improve access to medicines.^17^

Reducing medicines prices is high on government’s political agenda.^17^ Between 2012 and 2017, four medicine pricing policies were introduced, targeting the supply chain and incentives to medicines importers and local manufactures to improve access and contribute to improved health outcomes of Ghanaians. These policies include: (i) Health Commodity Supply Chain Master Plan (HCSCMP);^18^ (ii) framework contracting for medicines in high demand;^19 20^ (iii) Value Added Tax (VAT) exemptions for selected essential medicines^21 22^ and (iv) ring-fencing medicines for local manufacturing.^21^ Table 1 summarises key features of each policy.

**Table 1:** Summary of medicines pricing policies introduced between 2012 and 2017

Policy/year	Aim	Type of medicines	Pharmaceutical sector affected/beneficiary
HCSCMP (2012)	To address numerous challenges in the supply chain, for example, overlapping tasks, high costs and payment delays in procurement.	Essential medicines	Supply chain/public sector
Framework Contracting (2012)	To outline a centralised procurement process for bulk purchase and negotiation of medicine prices.	High demand essential medicines	Supply chain/public sector
VAT exemption for medicines importers (2017)	To remove build up costs due to taxes	392 selected essential medicines (imported finished products)	Importation/cost build-up due to taxes. Importers agreed to reduce prices of essential medicines by a minimum of 30%. Public and private sector
VAT exemption for active pharmaceutical inputs (API), manufacturing inputs and packaging materials (reviewed in 2012 and 2017)	To remove the build-up of costs due to taxes, and ring-fenced some selected essential medicines for local manufacturing	552 (active ingredients, and selected inputs) for essential medicines	Local manufacturing/cost build-up due to taxes Private

HCSCMP, Health Commodity Supply Chain Master Plan; VAT, Value Added Tax.

The HCSCMP sought to streamline procurement systems of essential medicines within the public sector, and was subsequently operationalised into the framework contracting, a strategy of HCSCMP which sought to address fragmented medicines procurement contracts.^18 19^ The two VAT exemption policies provided incentives to medicines importers and local manufacturers, respectively.

## Methods

We report results from a wider study which examined implementation of four medicine pricing policies in Ghana. Examining stakeholder roles, engagement and influences in the design and implementation of these policies was a critical component of the analysis and is the focus of this article.

### Conceptual framework

**Figure 1:**
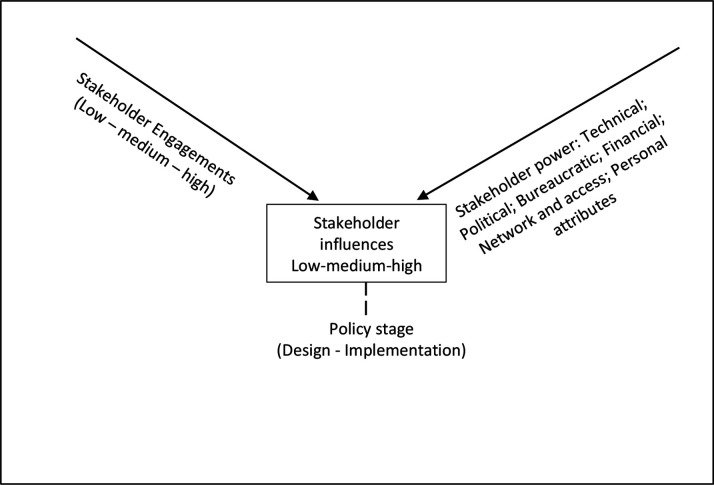
Theoretical framework (drawing upon).^23–26^

Our understanding of stakeholder engagements, powers and resultant influences over policy processes (figure 1) drew on the available conceptualisations of stakeholder powers, engagements and influences over health policy processes.^23–27^ Each stakeholder that is, actor with interest in a policy issue, has own sources of power which they can exercise to influence policy decisions as they engage in policy design and/or implementation.

The literature highlights six sources of stakeholder power: (a) *technical* that is, derived from knowledge, skills and information, (b) *political* that is, derived from political authority, (c) *bureaucratic* that is, derived from knowledge and authority of bureaucracies where policies are designed and implemented, (d) *financial* that is, derived from accessibility to financial resources, (e) *network* that is, derived from actors’ collective knowledge and action and (f) *personal attributes* that is, charismatic authority.^24^ Fundamentally, all stakeholders possess one or more sources of power, which they must exercise to be able to influence policy processes.^25^

Stakeholder engagements can be across the low–medium–high spectrum. Low degrees of engagement are when policy actors are *mere recipients of information* and are not actively involved in policy decisions. Medium degrees refer to *cases in which stakeholders are consulted to express their knowledge,* views and opinions. High degrees is when stakeholders had *actively participated, led or coproduced the policy design or implementation*.^23^ Stakeholder engagements arguably reflect their levels of interest, and willingness to participate, in the policy processes. Degrees of stakeholder engagement can also reflect spaces that stakeholders occupy at the policy table, such as provided (eg, policymakers), invited (eg, knowledge experts) and claimed (eg, advocacy coalitions).^7 26^ Thus, stakeholder powers, combined with engagements, inform stakeholder influences over policy processes. As with degrees of engagement, stakeholder influences can be along the low–medium–high spectrum,^10^ adapted to contexts of specific countries and policies.

Three points are worth noting on stakeholder influences. First, although more powerful stakeholders are more likely to exert greater influences over policies, these are subject to their engagements, which reflect their initial interests and willingness to participate in policymaking. For example, powerful stakeholders with expertise in the subject matter may be disinterested in a policy issue due to competing interest, resulting in their low engagement and consequently low influences. Second, stakeholder engagements require some power to exert influences over policy decisions. For example, knowledge experts with high interests and degrees of engagement can provide critical inputs when consulted in technical working grouping (TWG) forum to express their professional perspectives and thus are able to exert high influences over policy design and implementation decisions, perhaps similar to those who actively lead and implement those policies. Additionally, stakeholders with expertise and good understanding of the policy issue and are able to navigate the decision-making processes during TWGs and review meetings create opportunities to express their views and convince others to support their views to influence decisions.^28^ Conversely, some stakeholders views can be overlooked and ignored, despite their high interests and proactive engagements in policy processes. Furthermore, during policy design and implementation processes stakeholders who are not invited or do not claim space to participate in review meetings can miss the opportunity to express their views and consequently influence decisions. Third, sources of power, degrees of engagement and the resultant policy influences, can differ across the policy design and implementation. For example, frontline health workers may not be engaged in the policy design, but their actions essentially determine whether and how policies are implemented.^29^

### Data collection

We collected data using document reviews, in-depth interviews and consultative meetings with key policy actors.

We started by mapping policy actors and their roles from policy documentation, broadly defined as actual policies, regulatory documents and reports related to the design and implementation of the four policies (n=16). Our main inclusion criterion was relevance to the four policies, so documents covering other medicine pricing policies were excluded. Examples of specific documents included the Ghana supply chain assessment (2020), National Medicines Policy (2017), meeting minutes of the VAT exemption TWG (January 2018) and reports of VAT exemption implementation committee (February 2018). The documents were sourced from the MoH website (https://www.moh.gov.gh), Pharmacy Directorate and google scholar.

A total of 29 in-depth interviews (IDIs) were then conducted during August 2020–February 2021 (see table 2) by AK and LB. The respondents were purposefully identified from the documents and using snowballing from the IDIs and consultative meetings. Stakeholders who participated in policy decisions during policy design and implementation were considered. The IDIs sought to understand views and experiences of key policy actors in the development and implementation of four policies. The IDIs were conducted with national level policymakers, public and private health professionals, pharmaceutical wholesalers and importers and members of the National Medicines Pricing Committee (NMPC). The NMPC was inaugurated in 2019 to manage the medicine pricing system and comprises 22 members from the public and private sectors with direct or indirect interests in medicine pricing policies.

**Table 2:** List of respondents

Sector	Agency/institution	Number of respondents
Government agencies	Ghana Revenue Authority	1
	Ministry of Health	5
	Ministry of Finance	1
	National Health Insurance Authority (NHIA)	1
	Ghana Health Service Regional Health Directorate (GHS-RHD)	1
	Ghana Health Service Regional Medical Store (GHS-RMS)	1
	Ghana Health Service Headquarters (GHS-HQ)	2
Service providers	Teaching Hospital	1
	Regional Health Facility	1
	Public Hospital	3
	Public Polyclinic	1
	Private Hospital	1
	Christian Health Association of Ghana^72^	1
Development partner	WHO^33^	1
Professional association	Pharmaceutical Society of Ghana (PSGH)	1
	Society of Private Medical and Dental Practitioners	2
Pharmaceutical industry	Pharmaceutical Manufacturers Association of Ghana	2
	Community Pharmacy Practice Association (CPPA)	1
	Pharmaceutical Wholesaler/Importer/Retailer	1
NGO	Coalition of Non-Governmental Organisations in Health	1

NGO, Non-Governmental Organisations.

The IDIs utilised a question guide (online supplemental material 1), semi-structured by: framing of medicine pricing, roles of policy actors (expected and actual); implementation processes, approaches and timelines (anticipated and actual). The IDIs were conducted via telephone, zoom or in person as feasible and each was preceded by obtaining verbal or written informed consent. All IDIs were in English, lasted on average 45 min, were digitally recorded, transcribed verbatim and anonymised for analysis.

10.1136/bmjgh-2021-008225.supp1 Supplementary data

Five consultative meetings were held between October 2020 and February 2021, primarily to validate emerging findings but also to collect further data. These meetings involved purposefully identified NMCP members (October 2020, April 2021), pharmaceutical sector stakeholders (December 2020), medicine price mark-up working group (December 2020) and Society of Private Medical and Dental Practitioners (February 2021). The consultative meetings were an integral part of the study and were organised in collaboration with the MoH Pharmacy Directorate. The MoH invited attendees and the meeting averagely lasted 4 hours. During meetings, the MoH representatives and study members led discussions on the four policies and the meetings proceedings documented. To address potential recall bias, data sourced from interviews, document reviews and consultative meetings were triangulated.

### Data analysis

Data were analysed using thematic approach. The themes were organised according to the three dimensions from our conceptual framework (powers, engagements and influences) disaggregated by policy design and implementation. All authors participated in data analysis and interpretation after initial analysis by AK and TM. Emerging results were collated in tabular format for discussion and further analysis. The interview transcripts were the primary data sources for analysis, and insights from the documents and researcher notes from consultative meetings were used mainly to triangulate results from analysis of IDIs.

Our approach to determining low–medium–high degrees of stakeholder engagements and levels of influences has been informed by our analysis of data from the documents, IDIs and consultative meetings. In reporting our results and analysis, we primarily focused on stakeholders as organisations rather than individuals. This approach was driven by the emerging findings from the data, perhaps also reflecting the fluid and dynamic nature of policy processes (eg, with methodological questions of counting different individuals who were replacing their organisational colleagues in policy meetings, and those who were involved on one-off basis).

The study received approvals from ethics committees of the Ghana Health Service (GHS-ERC006/02/20) and the University of Leeds School of Medicine (MREC 19-060).

## Results

Individuals from 46 organisations, were identified from the documents, IDIs and consultative meetings, as key stakeholders with respect to the four policies (table 3).

**Table 3:** Stakeholder organisation

Sector	Stakeholder organisation	Abbreviation
Government agencies	**Ministry of Health**	MOH
	Minister for Health	Minister-MOH
	MOH-Procurement and Supplies Directorate	MOH-PS
	MOH-Pharmacy Department	MOH-PD
	MOH-Office of Chief Pharmacist	MOH-OCP
	MOH Ghana National Drugs P/rogramme	MOH-GNDP
	MOH-National Drug Information Resources Centre	MOH-NDIRC
	MOH-Central Medical Stores	MOH-CMS
	**Ghana Health Service**	GHS
	GHS-Regional Health Administrations	GHS-RHA
	GHS-Stores, Supplies and Drug Management	GHS-SSDM
	GHS-Regional Chief Pharmacist	GHS-RCP
	GHS-Regional Medical Stores	GHS-RMS
	GHS-Institutional Care Division	GHS-ICD
	GHS-Expanded Programme on Immunisation	GHS-EPI
	GHS-National Malaria Control Programme	GHS-NMCP
	GHS-National TB Control Programme	GHS-NTCP
	GHS-National AIDS Control Programme	GHS-NACP
	**National Health Insurance Authority**	NHIA
	NHIA-Provider Payment Directorate	NHIA-PPD
	Nurses and Midwives Council	NMC
	Pharmacy Council	PC
	Food and Drugs Authority	FDA
	Ministry of Finance	MOF
	Ghana Revenue Authority	GRA
	Ministry of Trade and Industry	MOTI
	Attorney General Department	AGD
	Public Procurement Authority	PPA
Service providers	**Public Service Providers**	
	GHS Health Facilities	GHS-HF
	Teaching Hospital Pharmacy Department	TH-PD
	**Private Service Providers**	
	Society of Private Medical and Dental Practitioners	SPMDP
	Christian Health Association of Ghana	CHAG
	Community Pharmacy Practice Association	CPPA
Development Partners	WHO^33^	WHO
	USAID|DELIVER project	DELIVER
	USAID|Global Health Supply Chain-Procurement and Supply Management	GHSC-PSM
	United Nations Industrial Development Organisation	UNIDO
Professional Associations	Pharmaceutical Society of Ghana	PSGH
	Ghana Medical Association	GMA
Pharmaceutical industry	Pharmaceutical Importers and Wholesalers Association	PIWA
	Association of Representatives of Ethical Pharmaceutical Institutions	AREPI
	Ghana National Chamber of Pharmacy	GNCOP
	Pharmaceutical Manufacturers Association of Ghana	PMAG
	Pharmaceutical Suppliers	PS
NGOs	Private Health Sector Alliance	PHSA
Politicians	Parliament Select Committee on Health	PSCoH
	Parliamentarians	Parliament
Patient groups	Cancer Connect Ghana	CCG
	National Diabetes Association	NDA

NGO, Non-Governmental Organisations.

**Figure 2:**
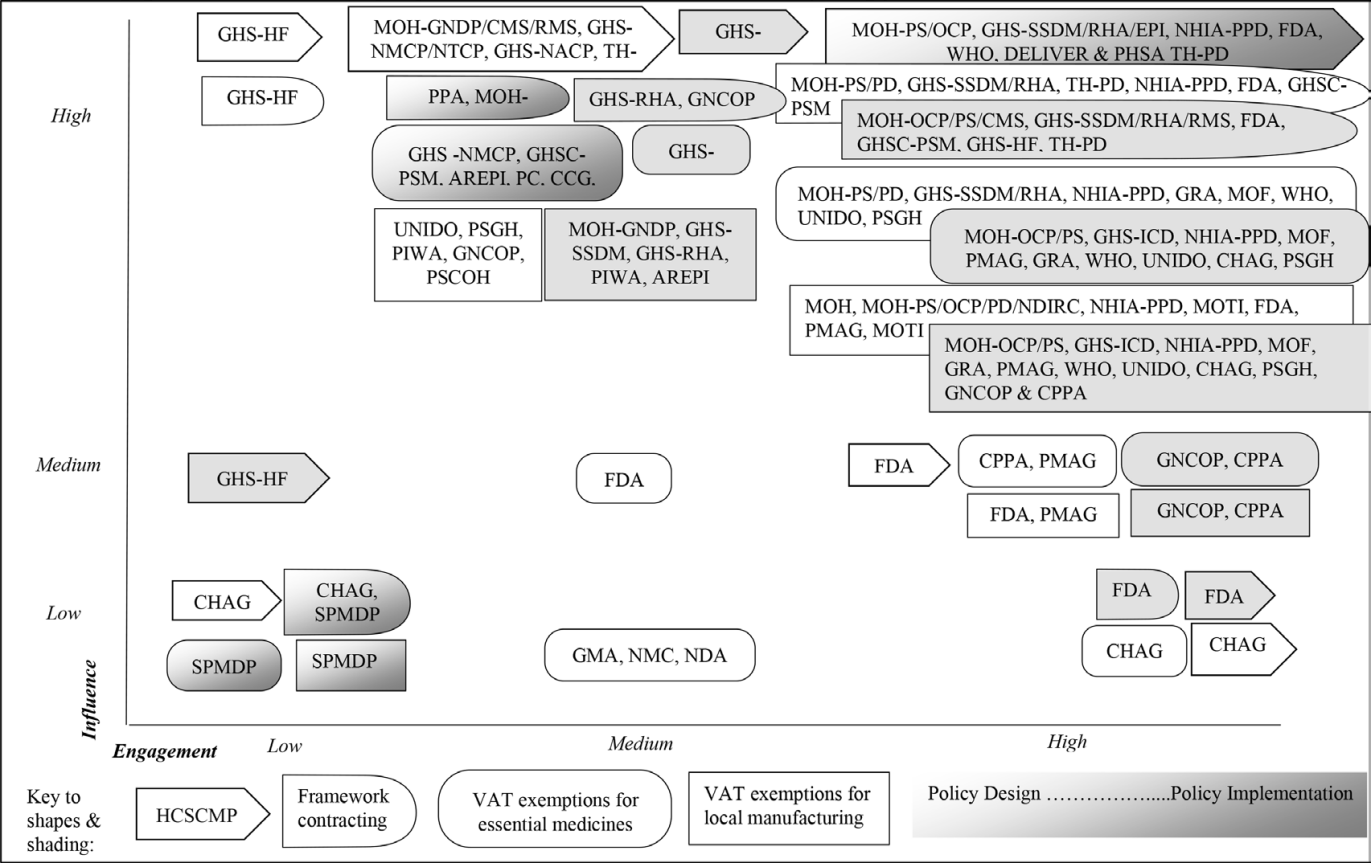
Engagements and influence by key stakeholders across the four medicines pricing policies.

Stakeholder roles, degrees of engagement, sources of power and resultant levels of influence in the design and implementation of the four policies are summarised in figure 2, tables 4 and 5 and are discussed next.

**Table 4:** Stakeholder roles and degrees of engagement in the design and implementation of medicine pricing policies in Ghana

Policy stage	Policy design		
*Stakeholder role/degrees engagement*	Low	Medium	High
Health commodity supply chain master plan (HCSCMP)	GHS-HF, CHAG—informed, supportive but not actively involved in the design process	MOH-GNDP, MOH-CMS, GHS-RMS, GHS-NMCP, GHS-NTCP, GHS-NACP, TH-PD—reviewed plans, participated in consultative meetings	MOH-PS—led and chaired Technical Working Group (TWG); drafted and reviewed plans and milestone MOH-PS, MOH-OCP, GHS-SSDM, GHS-RHA, GHS-EPI, NHIA-PPD, FDA, WHO, DELIVER and PHSA—drafted and reviewed plans and milestones; members of TWG DELIVER-technical advice, financial and secretarial support
Framework contracting for high demand medicines	GHS-HF, CHAG, SPMDP—informed but not involved in the design process	PPA—aware and supportive MOH-GNDP—reviewed medicine list	MOH-PS—led in identifying and developing the list of pharmaceuticals to be procured through a competitive tendering process MOH-PD, GHS-SSDM, GHS-RHA, TH-PD, NHIA-PPD, FDA and GHSC-PSM—participated in identifying and developing the list of pharmaceuticals to be procured through a competitive tendering process
Value Added Tax (VAT) exemptions for essential medicines	SPMDP—informed but not involved in the design process	GHS -NMCP, FDA, GHSC-PSM, AREPI, GMA, PC, NMC, CCG, NDA, PSCOH—reviewed the list of medicines for exemption and participated in stakeholder forum. AGD—drafted the policy into legislative content Parliamentarians—approved the list in parliament	MINISTER-MOH and MOH-PS—led the process and chaired the TWG MOH-PD, GHS-SSDM, GHS-RHA, NHIA-PPD, GRA, MOF, WHO, UNIDO, CHAG, PSGH, GNCOP, CPPA and PMAG—considered, agreed and recommended a list of selected imported pharmaceutical products for VAT exemption; members of the TWG MOH-NDIRC—secretarial support MOF—laid the Legislative Instrument before Parliament
VAT exemption for active pharmaceutical inputs (API), manufacturing inputs and packaging materials	SPMDP—informed but not involved in the design process	UNIDO, PSGH, PIWA, GNCOP, PSCOH—participated stakeholder meetings to build consensus Parliamentarians—approved the list in parliament AGD—drafted the policy into legislative content Parliamentarians—approved the list in parliament	MINISTER-MOH, MOH-PS, MOH-OCP and MOH-PD—led the process and chaired the TWG to review and update the list of restricted medicines and APIs for local manufacturing. MOH-NDIRC—secretarial support NHIA-PPD, MOTI, FDA, PMAG—reviewed and updated the restricted list of restricted medicines and APIs for local manufacturing. MOTI—approved the recommended list and participated in stakeholder meetings
*Policy stage*	Policy implementation		
*Stakeholder role/degrees engagement*	Low	Medium	High
Health commodity supply chain master plan (HCSCMP)	GHS-HF—implementing some aspect of the plans	GHS-RHA (non-ISC members), implementing aspects of the plan	MINISTER-MOH and MOH-PS—chaired the implementation steering committee (ISC) to develop and coordinate implementation modalities GHS-SSDM, GHS-RHA, MOH-CMS, GHS-RMS TH-PD, NHIA-PPD, FDA, WHO, DELIVER and PHSA-ISC members
Framework contracting for high demand medicines	SPMDP—informed but not implementing partner	PPA—evaluated tendering offers based on PPA guidelines, technical advice GHS-HF—submitted requests/demands to evaluation team GNCOP—participated in pre-bid conference	MOH-PS, GHS-RHA, TH-PD, NHIA-PPD—chaired tendering evaluation team, participated in the pre-bid conference and tendering process, reviewed and finalised the draft tender document MOH-OCP, GHS-SSDM, GHS-RHA, GHS-RMS, FDA, GHSC-PSM—members of tendering evaluation team. MINISTER-MOH—reviewed, approved and signed tender documents MINISTER-MOH-PS, GHS-RHA, MOH-CMS, GHS-RMS, GHS-HF, TH-PD—entered into a framework contract with the identified/successful suppliers
Value Added Tax (VAT) exemptions for essential medicines	SPMDP—informed but not implementing partner	MOH-GNDP, GHS-SSDM, GHS-RHA, AREPI—participated in stakeholder meetings to agree on modalities and methods of implementation	MINISTER-MOH-Deputy Minister of Health—approved implementation modalities and methods MOH-OCP—led the implementation technical working group (TWG) and coordinated implementation MOH-PS, GHS-ICD, NHIA-PPD, MOF, PMAG, GRA, WHO, UNIDO, CHAG, PSGH, GNCOP and CPPA—members of TWG
VAT exemption for active pharmaceutical inputs (API), manufacturing inputs and packaging materials	SPMDP—informed but not implementing partner	MOH-GNDP, GHS-SSDM, GHS-RHA, PIWA, AREPI—participated in stakeholder meetings to agree on modalities and methods of implementation	MINISTER-MOH-Deputy Minister of Health—approved implementation modalities and methods MOH-OCP—led the implementation technical working group (TWG) and coordinated implementation PMAG—implementing the policy MOH-PS, GHS-ICD, NHIA-PPD, MOF, GRA, PMAG, WHO, UNIDO, CHAG, PSGH, GNCOP and CPPA—members of TWG

**Table 5:** Stakeholders’ power sources and influence over the design and implementation of medicine pricing policies in Ghana

Policy/stage	HCSCMP			Framework contracting			VAT exemption for essential medicines			VAT exemption for local manufacturing		
Policy design												
Power/level of influence	Bureaucratic (approval)	Political	Technical	Bureaucratic (approval)	Political	Technical	Bureaucratic (approval)	Political	Technical	Bureaucratic (approval)	Political	Technical
Minister for Health	+++			+++			+++			+++		
MOH-Procurement and Supplies Directorate	+++		+++	+++			+++		+++	+++		
MOH-Office of Chief Pharmacist	++		+++							+++		
MOH-Pharmacy Department				+++			+++		+++	+++		
GHS-Stores, supplies and drug management			+++			+++			++			++
GHS-Expanded Programme on Immunisation			++									
GHS-Institutional Care Division									++			++
NHIA-Provider Payment Directorate			+++			+++			+++			+++
Food and Drugs Authority			++			+++			++			++
Ministry of Finance							+++		+++			+++
Ghana Revenue Authority							+++		+++			+++
Ministry of Trade and Industry												+++
Pharmaceutical Manufacturers Association of Ghana (PMAG)									++			+++
WHO			+++						+++			++
United Nations Industrial Development Organisation (UNIDO)									+++			+++
USAID|DELIVER			+++									
USAID|Global Health Supply Chain-Procurement and Supply Management						+++			++			
Private Health Sector Alliance			++									
Christian Health Association of Ghana^72^			+						++			++
Pharmaceutical Society of Ghana									+++			++
Ghana National Chamber of Pharmacy									+++			++
Community Pharmacy Practice Association (CPPA)									++			++
MOH Ghana National Drugs Programme			++			++			++			++
MOH Central Medical Stores			++									
MOH-National Drug Information Resources Centre									+++			+++
GHS-Regional Health Administrations			++			+++			++			+
GHS Regional Medical Stores			++						++			
GHS National TB Control Programme			+									
GHS National AIDS Control Programme			+									
Teaching hospital Pharmacy Department			++			+++						
GHS National Malaria Control Programme			+									
GHS Health facilities			+			++						
Pharmaceutical Importer and Wholesalers Association (PIWA)												++
Association of Representatives of Ethical Pharmaceutical Institutions (AREPI)									++			+
Ghana Medical Association (GMA)									+			+
Nurses and Midwives Council (NMC)									+			+
Pharmacy Council									++			++
Parliament Select Committee on Health/Parliamentarians								+++			+++	
Attorney General Department									+++			+++
Society of Private Medical and Dental Practitioners												
Public Procurement Authority						+++						
Cancer Connect Ghana									+			
National Diabetes Association									+			

Policy implementation												
Power/level of influence	Financial	Authority to implement	Authority to enforce, monitor and evaluate	Financial	Authority to implement	Authority to enforce, monitor and evaluate	Financial	Authority to implement	Authority to enforce, monitor and evaluate	Financial	Authority to implement	Authority to enforce, monitor and evaluate
Minister for Health	+++	+	+++	+++	+	+++	+++	+	+++	+++	+	+++
MOH-Procurement and Supplies Directorate	+++	+++	+++	+++	+++	+++		+++	+++		+++	++
MOH-Office of Chief Pharmacist		++	++									
MOH-Pharmacy Department		++	+++		++	+++		+++	+++		+++	+++
GHS- Stores, supplies and drug management		++	++		++	++						
GHS-Expanded Programme on Immunisation		++										
GHS-Institutional Care Division												
NHIA-Provider Payment Directorate		++			+++	+++		+++	+++		+++	+++
Food and Drugs Authority		+			+			+			+	
Ministry of Finance							+++	+++	+++	+++	+++	+++
Ghana Revenue Authority								+++	+++		+++	+++
Ministry of Trade and Industry											+++	+++
Pharmaceutical Manufacturers Association of Ghana (PMAG)										+++	+++	+++
WHO		+	++						+++			
United Nations Industrial Development Organisation (UNIDO)												
USAID |DELIVER	++	++	+++									
USAID|Global Health Supply Chain-Procurement and Supply Management				+++	++	+++						
Private Health Sector Alliance			+									
Christian Health Association of Ghana^72^												
Pharmaceutical Society of Ghana											++	
Ghana National Chamber of Pharmacy					++	++	+++	+++	+++	++	+	++
Community Pharmacy Practice Association (CPPA)								++				
MOH Ghana National Drugs Programme						+			+			+
MOH Central Medical Stores		++	+		+++	+						
MOH- National Drug Information Resources Centre									+			+
GHS-Regional Health Administrations		+++	+++		+++	++						
GHS Regional Medical Stores		++	+		+++	+						
GHS National TB Control Programme												
GHS National AIDS Control Programme												
Teaching hospital Pharmacy Department		+++	+		+++	++						
GHS National Malaria Control Programme												
GHS Health facilities		+++	+		+++	+						
Pharmaceutical Importer and Wholesalers Association (PIWA)								+++	+++			
Association of Representatives of Ethical Pharmaceutical Institutions (AREPI)								+++	+++			
Ghana Medical Association (GMA)												
Nurses and Midwives Council (NMC)												
Pharmacy Council												
Parliament Select Committee on Health/ Parliamentarians												
Pharmaceutical Suppliers					+++	+		+++			++	
Attorney General Department												
Society of Private Medical and Dental Practitioners												
Public Procurement Authority					+++	+						
Cancer Connect Ghana												
National Diabetes Association												

Notes:+++, high influence; ++, medium influence; +, low influence; empty cells—no influence identified.

HCSCMP, Health Commodity Supply Chain Master Plan; VAT, Value Added Tax.

Most stakeholders had high–medium engagements (26/46, 56.5% and 27*/*46, 58.7%, respectively) and very few (3/46, 6.5%) had low engagements. The most highly engaged stakeholders were individuals from the MoH, other government agencies, development partners, pharmaceutical industry and professional bodies. The least engaged stakeholders were some service providers.

Two mechanisms were used by the MoH to facilitate stakeholder engagement, which contributed to inclusive and participatory policy development and implementation. First, eight TWGs were established by the MoH Procurement and Supplies, and Pharmacy directorates, for design and implementation of each policy. The TWGs met on average once a week for 2 months and collectively drafted, reviewed and coproduced plans and milestones, and implementation strategies. Some stakeholders were part of both the design and the implementation TWGs, while others were involved in one only. Stakeholder involvement in the TWGs reflected their technical expertise, roles, political, bureaucratic and financial mandates and interests. Second, the MoH conducted extensive stakeholder consultations. These involved meetings with a range of stakeholders to share knowledge, views, opinions and … to finalise the policies and how to ensure that the policies are implemented as intended (Governmental Officer). These meetings lasted typically 0.5–1 day, with the TWG members presenting plans for review and discussion.

Next, we report powers, engagements and resultant influences by five main stakeholder groups which emerged from our analysis.

### Health sector agencies

Two MoH directorates featured prominently across all four policies: the Procurement and Supplies, and the Pharmacy. They formed, and actively engaged in, all TWGs, reflecting their technical expertise, high interest, mandates and high influence over policy design and implementation, which ultimately contributed to inclusive and participatory policy development and implementation:

National medicine pricing policies are initiated by government through the MoH, and we have all come to accept that. (Professional association)

The Procurement and Supply directorate led the design and implementation of the pooled procurement, tendering and negotiations of medicine pricing in HCSCMP and framework contracting, whereas the Pharmacy directorate led the design and implementation of the VAT exemption for selected essential medicines and local manufacturing. However, the Pharmacy directorate exerted medium influence over the implementation of the HCSCMP and the Framework contracting, since implementation of pooled procurement policies was outside their remit.

The GHS had representatives in all TWGs except in the design of VAT exemptions for local manufacturing. This reflected their mandate, for example that supporting manufactures and providing technical expertise was not a GHS’s core mandate. The following GHS representatives were highly engaged and contributed expertise in supply chain management in the TWGs for the HCSCMP and framework contracting policy processes: Supplies, Stores and Drug Management (SSDM) directorate (GHS headquarters) and the Regional Health Administrations (RHAs) for Eastern, Central and Western regions. The RHAs controlled implementation structures at peripheral level and consequently had high influence over implementation of these policies. This allowed for local ownership of policy implementation and therefore contributed to implementation feasibility within local context. However, the SSDM at the GHS headquarters with oversight role on implementation had medium influence over actual policy implementation.

Meanwhile, the Institutional Care division (GHS headquarters) was highly engaged only in the implementation TWGs for VAT exemptions for essential medicines and local manufacturing, but with no reported influence on policy implementation. On the other hand, selected GHS health facilities who were implementing framework contracting, engaged with the TWG on their region-specific requests during the pre-bidding conference, and as implementers wielded high influences over policy implementation by ensuring that implementation is grounded within realities of their local organisational and cultural contexts.

Document review also highlighted that the representatives from the GHS programmes for TB, Malaria and AIDS and the Regional Medical Stores were engaged in a consultative meeting to review the HCSCMP implementation but exerted low influences due to their disease-specific focus and medical stores activities.

As far as I know, all major players were involved. The GHS a government agency was involved, and also other provider groups including CHAG, pharmaceutical supplies, and community pharmacies. (Industry association)

The NHIA with interest in access to affordable essential medicines was highly engaged in TWGs across all four policies. Its provider payment directorate drew on their experiences in negotiating medicine prices and exerted high influences over the implementation of framework contracting and VAT exemptions policies, for example through chairing tenders for framework contracting creating the opportunity to share experiences and expertise and through this making the process more rigorous. Similarly, a representative from the Korle-Bu Teaching Hospital’s Pharmacy Department with high interest in affordable medicines and expertise in tendering, chaired one of tendering evaluation processes for framework contracting creating the opportunity to collate and synthesise other views and perspectives and also contributing to policy implementation inclusiveness. Two other teaching hospitals (Cape Coast and Tamale) were also highly engaged in the TWGs for framework contracting and contributed experiences in bulk purchasing and price negotiations.

A representative from the Central Medical Store (CMS) with technical expertise and authority to implement had medium influence over implementation of the HCSCMP across the peripherals. On the other hand, an individual from the National Drug Information Resource Centre used technical skills to provide secretariat support for the VAT exemptions TWGs and had control over the document wording exerting high influence in the design and implementation of the VAT exemptions.

A broader range of stakeholders were interested and engaged in the consultative meetings on the VAT exemptions policies, than the HCSCMP and framework contracting, reflecting multisectoral nature of the VAT exemptions. While that meant more inclusive policy processes with more views and perspectives been considered that also meant greater number of consultations to harness the diverse views and perspectives. These stakeholders included two MoH regulatory agencies (Pharmacy Council and Nurses and Midwife Council); Ghana Medical Association, which represents physicians, surgeons and dentists; and two patient groups (Cancer Connect Ghana and National Diabetes Association). Although consulted based on their technical expertise, interviews showed that these stakeholders had low influences on the implementation of the VAT exemption policies, perhaps reflecting their limited political and bureaucratic powers.

### Government agencies

The MoH closely engaged other interested government ministries in all policies, through inviting them to the TWGs and stakeholder consultation meetings. This reflected the MoH’s desire for participatory policymaking, and enabled leveraging bureaucratic, political and technical powers of government agencies to ensure inclusion of multiple stakeholder views and perspectives during policy development and implementation.

For consensus building and common understanding, the MoH organised meetings bringing all stakeholders on board including representatives from other ministries. (Health policymaker)

The high-level government stakeholders with political powers highly influenced policy design, but not the implementation. The Parliamentary Select Committee on Health, using its legislative mandate, reviewed and approved the VAT exempted medicines list and the Parliament of Ghana with legislative power passed the Law on VAT exemptions.^30^ While both exerted high influences over policy design, neither influenced policy implementation reflecting their disinterest in policy implementation. Similarly, the Attorney General Department (AGD), a legislative drafting session of the Office of the Attorney General and Ministry of Justice, provided legal advice in drafting the VAT exemptions policies into a legislative Bill for Parliament to approve. The resulting regulation^30^ is the law that guides the implementation of VAT exemptions for medicines. While the AGD highly influenced the design of VAT policies, they did not influence policy implementation. Similarly, the Food and Drugs Authority (FDA) was highly engaged as TWGs member for the HCSCMP and framework contracting but had reportedly low influence over their implementations. This is because their TWG members provided technical advice on medicines quality, registration status and local manufacturing capacity, but were not involved in policy implementation.

We found examples of government agencies influencing both policy design and implementation. The Ministry of Finance (MoF) and the Ghana Revenue Agency (GRA) were highly engaged as TWG members and advised on the design of 30% reduction in medicines prices, cost build-up for wholesalers and manufacturers and establishing average period for VAT exemption approval.^22^ During implementation, the MoF and GRA who were involved in the design of implementation modalities were processing applications for VAT exemptions for imported essential medicines and restricted medicines for local manufacturing, and thus were enforcing, financing, monitoring and evaluating VAT exemptions policies. This allowed for consistent key stakeholder ownership across the policy design and implementation and consequently contributing to feasibility of policy activities within national context.

Our results also reveal two examples of government agencies influencing policy implementation only. First, the Ministry of Trade and Industry (MOTI), which also promotes private sector development, was in TWG for the VAT exemptions for local manufacturing and highly influenced policy implementation because of their political and bureaucratic powers. The MOTI advocated for a list of restricted medicine that would protect the interest of the patients, wholesalers, importers and local manufacturers, thus contributing to more equitable policies that is, consideration of benefits from these policies to wide range of stakeholders. Second, the Public Procurement Authority (PPA), a state organisation with a mandate to ensure efficient and transparent public procurement, was highly engaged in framework contracting and reviewed the tender offers against their guidelines.^31^ Consequently, it wielded high influence over the implementation of the framework contracting policy contributing to a transparent and accountable implementation modalities.

### Development partners

The interviews and TWG meeting minutes showed that roles of three developmental partners were spread across the policies, reflected their technical expertise and interests.

You know the donors have certain areas that they focus on. For example, the USAID projects were interested in procurement related policies. (Industry Association)

The USAID-funded projects supported procurement, logistics and supply chain management.^32^ For example, representatives from the DELIVER project and Global Health Supply Chain-Procurement and Supply Management (GHSC-PSM) were highly engaged as TWG members for HCSCMP and framework contracting, respectively. They provided technical advice, financial and secretarial support and as such highly influenced feasibility and technical rigour of policy design and implementation coproducing policy content. On the other hand, the GHSC-PSM also reviewed implementation modalities for VAT exemptions for essential medicines but had no reported influence over their implementation.

The UNIDO engaged in the design and implementation of the VAT exemptions for essential medicines and was a TWG member for the design of VAT exemptions for local manufacturing, providing economic and industrial advice. Through their expertise and knowledge, the UNIDO representative participated in the coproduction of the policy content wielding high influence over the technical rigour of the policy design but had no influence over policy implementation.

The representative from WHO^33^ provided technical advice for HCSCMP and both VAT exemptions policies (implementation only) wielding high influence over evidence-informed nature of policy design by sharing guides on access to medicines and other countries’ examples, but with low-to-medium influence over policy implementations. The WHO also provided general support to health policymaking, for example the design of the National Medicine Policy, the National Standard Treatment Guidelines and Essential Medicines Lists.^34–36^

### Industry and professional associations

These were highly engaged by the MoH in the design and implementation of all policies.

The MoH involved the pharmaceutical sector players for their buy in so that as they participate in the drafting of the policies, they will accept its implementation in good faith and ensure reduction in medicine prices. (Governmental Officer)

However, professional associations had varying influences over policies, with most notable influences exerted over two VAT exemption policies. Their involvement reflected level of interest and differing roles in policy design and implementation, as we explain next.

The representatives from Pharmaceutical Society of Ghana (PSGH), Ghana National Chamber of Pharmacy, Community Pharmacy Practice Association (CPPA) and Pharmaceutical Manufacturers Association of Ghana (PMAG) with high interest contributed professional expertise as TWG members for the design and implementation of the VAT exemptions. The PSGH exerted medium influence over implementation of the VAT exemptions for local manufacturing, as a body with monitoring role over the pharmacy practices including manufacturing of pharmaceuticals. The CPPA, representing the community pharmacies, exerted high influence over the technical rigour and feasibility of design and implementation of the VAT exemptions for essential medicines, drawing on their experience in designing and implementing mark-up regimes. However, they had no identified influence over implementation of the VAT exemptions for local manufacturing.

The Association of Representatives of Ethical Pharmaceutical Institutions (AREPI) and Pharmaceutical Importers and Wholesalers Association (PIWA), as policy implementers representing wholesalers and importers, wielded medium influences over policy contents as they reviewed the VAT exemptions for selected essential medicines and local manufacturing. The Ghana Chamber of Pharmacy, however, reviewed only plans for VAT exemption for local manufacturing and had low influence over policy implementation reflecting their different focus. Although framework contracting was implemented through the public health facilities,^18^ the pharmaceutical suppliers and Ghana National Chamber of Pharmacy were engaged in, and influenced, policy implementation.

### Service providers

These stakeholders, even with technical skills, interest and technical expertise, were not specifically invited, engaged and did not claim space to influence the four policies. Although we do not have data that directly explain this, it is likely to be due to their limited bureaucratic and political powers in relation to national-level medicines pricing. The Society of Private Medical and Dental Practitioners (SPMDP) was not actively involved in any policies, neither as a group nor as individuals. Although the policies were designed to reduce medicines prices and improve access within public and private sectors, the SPMDP did not contribute their expertise and experiences.

The private health facilities were not involved and has not benefited from implementation of these medicine pricing policies. (Professional Association)

Representatives of some GHS health facilities who were interviewed were unaware of the implementation modalities of the four policies. As noted by one respondent:

I work in the public sector and in a district hospital, but I was not involved in any other discussions or stakeholder engagements. (Service Provider).

CHAG, which represents 344 faith-based facilities,^37^ were not involved in the design of the HCSCMP and framework contracting. A CHAG member implemented a pooled procurement programme in 2012,^38^ but their expertise was not considered. The CHAG representatives were highly engaged in the VAT exemptions policies, but with no visible influences over policy implementation.

## Discussion

We reported analysis of stakeholder roles, engagements, powers and resultant influences over design and implementation of four medicines pricing policies in Ghana. In doing so, we also highlighted the critical role of stakeholder analysis in understanding, informing and improving health policy processes.^8–11^

Five out of six sources of power^24^ featured in our results: technical (expertise of meeting contributors and chairs), political (legislative authority to approve policy), bureaucratic (authority to formulate, implement, enforce, monitor and evaluate policies), financial (access to/control over budgets) and network (collective power within TWGs). Personal characteristics did not feature explicitly, suggesting that stakeholders were primarily understood as organisations and not as individuals, contrasting the literature on individual charismatic policy champions.^39–43^ Our results also reveal closed and invited policy spaces and visible powers, with no claimed spaces or hidden or invisible powers.^7 26^ This may reflect a strong MoH coordination role but may also reinforce methodological challenges of identifying these phenomena. Our results also reinforce the importance of identifying specific manifestations of power,^24–26^ such as authority to formulate or implement within bureaucratic power.

All four policies had high-to-medium stakeholder engagements in their design and implementation. The high engagements by non-health stakeholders in the VAT exemption policies highlight the importance of multisectoral approaches to medicines pricing^18 19 22^ and broader health policymaking.^10 12^ By the virtue of their mandate, influence and interests, policymakers, pharmaceutical industry and developmental partners played prominent roles. This is consistent with earlier-reported similar roles of policymakers,^44^ pharmaceutical manufactures,^45^ wholesalers and retailers,^46 47^ private sector and other state actors.^48–52^

Stakeholder engagements, combined with the exercise of power, determine their influences over policy processes,^10 24^ such as inclusive, participatory and evidence-informed policy development or feasible implementation grounded within local contexts. However, a mere existence of power alone is not sufficient and requires adequate engagements to influence policy decisions, illustrated by powerful government officials and development partners only engaging in some policies reflecting their mandates and interests. Similarly, high degree of engagements by less powerful stakeholders may not result in policy influence, as exemplified by the high engagements of the GHS-SSDM in both HCSCMP and the VAT exemptions policies but with different influences over each policy.

Two MoH directorates led all four policies by establishing TWGs and facilitated stakeholder consultations. Such an approach can facilitate opening up a ‘decision space’^53^ and ensure inclusive, transparent and evidence-informed policymaking. Our findings echo the Thailand’s experience where Ministry of Public Health as ‘progressive bureaucrats’ also engaged others in the UHC reforms.^54^ However, policymakers require adequate capacity to ensure availability of policy spaces and avoid omissions of stakeholders with relevant expertise,^26 55 56^ as was the case with the exclusion of CHAG from HCSCMP and framework contracting policies in our study. Prominent roles of development partners within the Ghanaian health sector^57^ mirror other LMICs,^58^ and also reinforce the importance of strong leadership by the national policymakers.^55 57 59 60^

Limited awareness of the four policies by the grassroots-level health service providers in Ghana is worth noting. It may reflect insufficient communication by the policymakers but may also be due to limited information sharing by the TWG members within their organisations and networks. If unaddressed, limited local awareness can constrain engagements, and contribute to lack of ownership, detachment, and fuel resentment towards policy implementation.^29 61^

The nature of the policy issue and the approach to policy design and implementation^42 62–65^ appear to have influenced stakeholder engagements and interest in the four policies. For example, minimal engagements from the private sector in the implementations of the HCSCMP and the framework contracting policies (such as SPMDP despite them being beneficiaries of both policies) was because the implementations of these policies were done primarily through the public sector and perhaps through more enclosed TWGs as policy networks.^66 67^ In contrast, the multisectoral VAT exemptions policies had engagements and interest from a wider range of stakeholders.

Our results helped to understand stakeholder roles within the four policies, and also provided deeper insights into the determinants of health policy processes more generally. Robust health policy processes are typically understood as being participatory, inclusive, clear, transparent and evidence-informed.^55 64^ All these characteristics are affected by degrees of stakeholder engagement, power dynamics and the resultant influences on policy processes.^6 24 43 68 69^ Adequate capacity of national policymakers for strong leadership and coordination of health policy processes is particularly important.^42 55 57 70 71^

Our results suggest four policy implications and lessons for improving the medicine pricing policy processes in Ghana and similar contexts. First, identifying who has a stake in issues of pricing, availability and affordability of medicines and the process of generating an inclusive list of stakeholders is a critical step in ensuring participatory policymaking. Second, considering stakeholder experiences, perspectives, needs, interest and expectations are critical steps in reducing gaps between the policy intent and actual implementation. Third, the nature of a policy issue and the approach to policy design and implementation can enable or constrain stakeholder involvements in policy processes, and consequently their inputs into decision-making. Finally, given a complex and time-consuming nature of policymaking, there is a need to identify the most feasible and context-specific ways of ensuring adequate stakeholder engagements within policy processes.

We acknowledge four study limitations. First, we mainly analysed stakeholder roles within the four policies. While we also reported key effects on policy processes, a more detailed analysis of policy processes was outside the primary scope of our paper and represents an agenda for future research. Second, the views of representatives in TWGs may not be generalisable to their respective organisations, and a larger study may be appropriate where resources permit. Third, implementations of four policies are ongoing, so stakeholder powers, roles, interests and engagements and resultant influences may change. While this highlights a more general limitation of stakeholder analysis, it also provides an opportunity for follow-on analyses to compare trends over time. Finally, documented evidence on stakeholder powers were understandably limited, and our strategy was to draw on data from the interviews and consultive meetings. Despite these limitations, our analysis should provide useful information for improving stakeholder involvements in the design and implementation of medicine pricing policies in Ghana and beyond.

## Conclusion

Effective leadership is important for ensuring inclusive and participatory policymaking. Policymakers and analysts should also be cognisant of the nature of policy issues and approaches to policy design and implementation. Current and future medicines pricing policies can be improved by identifying key stakeholders and considering their experiences, perspectives, needs and expectations; greater understanding of the nature of a policy issue and approaches to policy design and implementation, and their implications on stakeholder engagements and developing feasible and context-specific ways of enhancing stakeholder engagements within complex policy processes.

## Data Availability

All data relevant to the study are included in the article or uploaded as supplementary information.
