# Supplementary material for: Powers, engagements and resultant influences over the design and implementation of medicine pricing policies in Ghana
Source: BMJ Glob Health. 2022 May 18;7(5):e008225. doi: 10.1136/bmjgh-2021-008225 (PMC9121428; doi:10.1136/bmjgh-2021-008225)
Supplement: Supplementary data [file bmjgh-2021-008225supp001.pdf]

Supplementary Material 1: Interview guide

Participant information sheet and consent form will be sent to respondents and consent sought before conducting the interview.

The medicine pricing policies for our discussions are:
1. Health commodity supply chain master plan (HCSCMP)
2. Framework contracting for high demand essential medicines
3. Value Added Tax (VAT) exemptions for essential medicines
4. VAT exemption for active pharmaceutical inputs (API), manufacturing inputs and packaging materials

I. Introduction to respondents

1. Tell me about your organization and its areas of focus and role in the health sector?
2. Tell me about your role in the organisation and your specific areas of focus in relation to access to essential medicines?

II. The medicine pricing problem definition and policy design [for each medicine pricing policy]

1. How was the problem of medicine pricing defined and framed, by which stakeholders and why?
2. What medicines are targeted and why? (Medicine category, population subgroup targeted)

III. Implementation approaches [for each medicine pricing policy]

1. Which implementation processes, mechanisms and timelines were anticipated, why and by whom? In your opinion were these implementation processes, mechanisms, and timelines appropriate and what would you recommend moving forward?
2. How are the policies currently implemented (in relation to implementation processes, steps, resources allocated, timing, involvement of actors, role of evidence etc) and why?
3. Which stakeholders were expected to engage in policy implementation, how and in what capacities or roles and why? How does this compare with any evidence of actual stakeholder involvements? What is your role in implementation process? Who else should be added and why? Were there stakeholders who opposed the implementation of these policies and why?
